# NPP-Digital Psychiatry and Neuroscience (DPN): a new journal for the era upon us

**DOI:** 10.1038/s44277-023-00001-6

**Published:** 2023-10-17

**Authors:** William A. Carlezon, Sarah Timm

**Affiliations:** 1 Department of Psychiatry, Harvard Medical School, McLean Hospital, Belmont, MA 02478 USA; 2 American College of Neuropsychopharmacology, Brentwood, TN 37027 USA

**Keywords:** Biomarkers, Signs and symptoms

We are delighted to launch NPP-Digital Psychiatry and Neuroscience (DPN) as an official journal of the American College of Neuropsychopharmacology (ACNP). A sibling of ACNP’s flagship journal *Neuropsychopharmacology* (NPP), DPN is Open Access and online-only, with rolling publication of accepted papers, and organized by collections rather than issues. While functionally separate journals, DPN and NPP will work together to provide authors with venues that are optimized for specific content and have a high impact upon readership.

Rapid advances in technology are changing ways in which psychiatric illness is diagnosed, treated, and studied. Accordingly, DPN has two primary goals, both of which are evident in the journal name. One goal is to focus on research that involves the use of digital technologies—including (but not restricted to) smart devices, wireless telemetry, motion sequencing, artificial intelligence, machine learning, bioinformatics, and computational approaches—in the diagnosis, treatment, prevention, and study of psychiatric illnesses. A second but equally important goal is to better align psychiatry and neuroscience, enabling more effective and impactful interactions between clinical care and basic (i.e., neuroscience, neurobiology) research. In humans, continued refinements in brain imaging, increasing utilization of smart devices and wearables, and advances in the efficiency of genetic tools are among developments that have enabled fundamentally new insights into healthy and aberrant brain function, as well as molecular factors that contribute to vulnerability to illness. In animal model systems, there has been a corresponding evolution of precision molecular techniques to probe and dissect the neural circuitry underlying complex behavior. Despite these advances, psychiatry and basic neuroscience continue to evolve largely in parallel, with few instances where the fields have aligned to produce transformative improvements in human health. DPN will endeavor to identify areas where these fields connect; borrowing an electrical term, we seek reports that provide an “arc”—an energetic connection between parallel electrodes that creates a luminous discharge. In the context of mental health research, these types of connections are increasingly facilitated by digital, big-data approaches.

With respect to scope, DPN will specialize in articles that focus on psychiatric disorders, as opposed to those that focus on other brain conditions that sometimes feature comorbid psychiatric symptoms (e.g., Parkinson’s Disease). Successful reports will have three main attributes. Foremost, DPN will prioritize the use of endpoints that are translationally-relevant, meaning that they are similar or virtually identical in humans and other species used to study psychiatric illness. Prototypical examples of endpoints include those that can be derived from smartphones and wearables (e.g., sleep, body temperature, activity, heart rate); performance in translationally-aligned (e.g., touchscreen) behavioral tasks that quantify attention, motivation, or threat; blood-borne markers; and/or gene expression patterns. A second critical attribute is the use of continuous data collection, where endpoints are measured over sustained periods (hours, days, or longer). The analogy here is moving from snapshots to film, thereby enabling more comprehensive data interpretation that is less dependent on one-time, arbitrary factors. A third critical attribute is use of data collection and analysis techniques that are objective, reproducible, and inter-rater reliable, and that minimize subjective judgements, particularly in the context of attributing complex human traits or feelings to laboratory animals. Considering these priorities, DPN is far more likely to publish a paper on the effects of stress in mice on heart rate variability (a cross-species endpoint that involves continuous measurement by a device) than on time struggling in the tail suspension test (an endpoint measured at a restricted time point in a procedure with no human equivalent, thereby requiring anthropomorphic interpretations). It is important to note that some techniques (e.g., brain imaging) fulfill all of these attributes but involve substantial procedural deviations (e.g., restraint or anesthetic) or fundamental differences in capabilities (e.g., ability to understand instructions, guidance, or reassurance) across species; as such, manuscripts will not automatically qualify for consideration at DPN simply because they involve a digital aspect.

DPN is led by a team of Senior Editors who have been at the forefront of advances including the use of digital phenotyping—continuous assessments of behavioral patterns via devices and/or tracking systems—in humans and laboratory animals [1–3], social media for research on mental health [4], and big-data approaches to analyze gene function [5]. The team has experience with using journal capabilities creatively, as community resources, to promote diversity, equity, participation, and outreach in ways that resonate with scientific and non-scientific audiences [6, 7]. We will also have an Editorial Internship program that offers perspective on the daily operations involved in running a scientific journal. Each Senior Editor has selected a small team of Editorial Board Members to provide insight as the journal establishes operations. Over time, these teams will grow in size and scope, as the journal expands into new areas driven by developments in psychiatry and neuroscience. Inside the journal, DPN will have special features that reflect our aspirations to think creatively about the types of content will be maximally useful to our readership. As one example, we will have a new article-type called “Didactics”, which is intentionally brief and designed to introduce important psychiatric principles to neuroscientists, and important basic research concepts to psychiatrists. To promote diversity, we have devised processes to encourage authors to be mindful in their selection of which citations to include in their article; to promote outreach, we will require that each accepted article include a lay-person summary that is designed to help the public understand the impact of the work. We hope that our embrace of these features will have a positive impact, beyond traditional journal metrics, in ways that inspire both scientists and lay persons and add value to the articles that we publish.

Overall, DPN’s activities will align with those of the ACNP, its parent organization. The ACNP’s mission is to advance scientific understanding of, and to facilitate communication about, disorders of the brain and behavior in order to advance their prevention and treatment. Inseparable from these scientific and medical priorities is ACNP’s commitment to teaching, diversity, outreach, and citizenship. Both its flagship journal (NPP) and annual meeting are renowned for cutting-edge science and forward thinking, and DPN will continue this legacy by showcasing the continued innovation that is emerging in its namesake disciplines while being mindful of the communities in which we exist. Indeed, the ACNP has even committed to being “carbon negative”—a paradoxically positive term whereby the organization offsets >100% of its annual meeting carbon footprint via donations to organizations selected for low overhead and maximal impact. The fact that DPN is an open-access and purely online journal, with no print versions to manufacture and distribute across the globe, is consistent with these commitments.

The journal name represents our best efforts to use terms that are inclusive of the most modern and exciting approaches in the field of brain research right now. We acknowledge that still-to-be-defined frontiers extending beyond digital are likely just around the corner, along with transformative advances in our understanding of how non-neuronal brain cells and peripheral organs contribute to nervous system function. We envision DPN as an easily-accessible, shared resource that enables the mental health and neuroscience communities to discover, learn, and teach together. We hope to be a unique resource as “digital” rapidly becomes an integral element of the field where psychiatry and neuroscience intersect, and to become a leading venue for researchers operating in this domain.

## Citation diversity statement

The authors have attested that they made efforts to be mindful of diversity in selecting the citations used in this article.
